# Supplementary material for: Transcriptotype-Driven Discovery of Apigenin as a Therapy against Cholestatic Liver Fibrosis: Through Inhibition of PANoptosis and Following Type-I Interferon Responses
Source: Antioxidants (Basel). 2024 Feb 20;13(3):256. doi: 10.3390/antiox13030256 (PMC10967634; doi:10.3390/antiox13030256)
Supplement: Supplementary file 1 [file antioxidants-13-00256-s001.zip › antioxidants-2738709-supplementary.pdf]

Supplementary material

Supplementary figures and legends

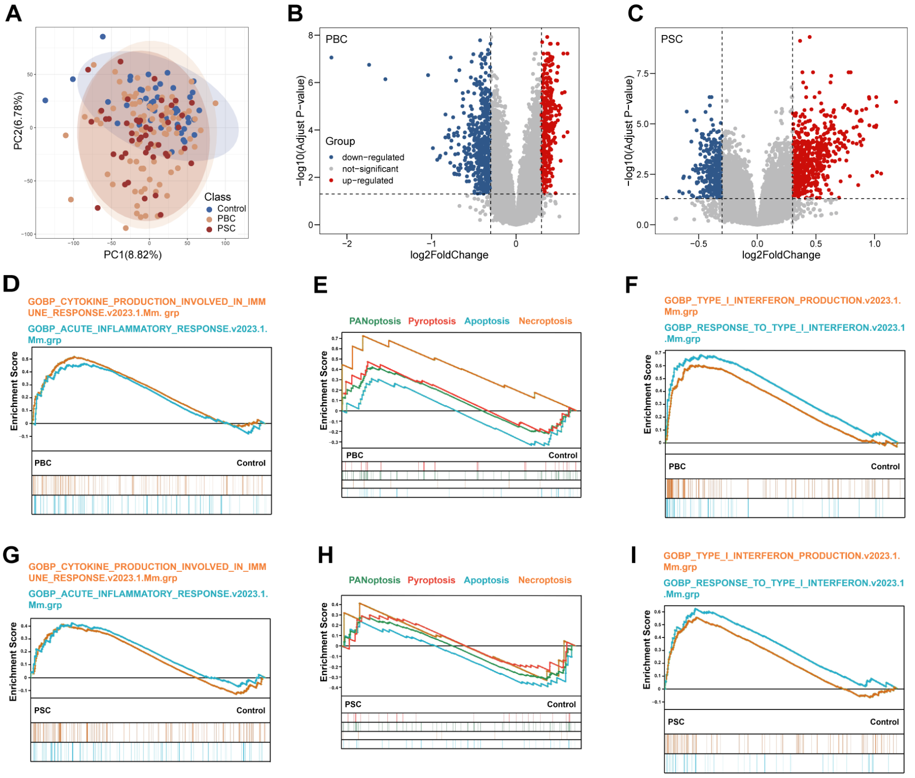

Figure S1. Analysis of published RNA-seq data (GSE 119600) with 90 PBC patients and 45 PSC patients, and 47 healthy controls. (A) PCA plot of the RNA-seq results. **(B)** Volcano plot of DEGs in blood of PBC patients and healthy controls. **(C)** Volcano plot of DEGs in blood of PSC patients and healthy controls. **(D)** GSEA of gene sets for cytokine production involved in immune response and acute inflammatory response pathway between PBC patients and healthy controls. **(E)** GSEA of gene sets for PANoptosis, pyroptosis, apoptosis and necroptosis pathway between PBC patients and healthy controls. **(F)** GSEA of gene sets for type-I IFN production and response to type-I IFN pathway between PBC patients and healthy controls. **(G)** GSEA of gene sets for cytokine production involved in immune response and acute inflammatory response pathway between PSC patients and healthy controls. **(H)** GSEA of gene sets for PANoptosis, pyroptosis, apoptosis and necroptosis pathway between PSC patients and healthy controls. **(I)** GSEA of gene sets for type-I IFN production and response to type-I IFN pathway between PSC patients and healthy controls.

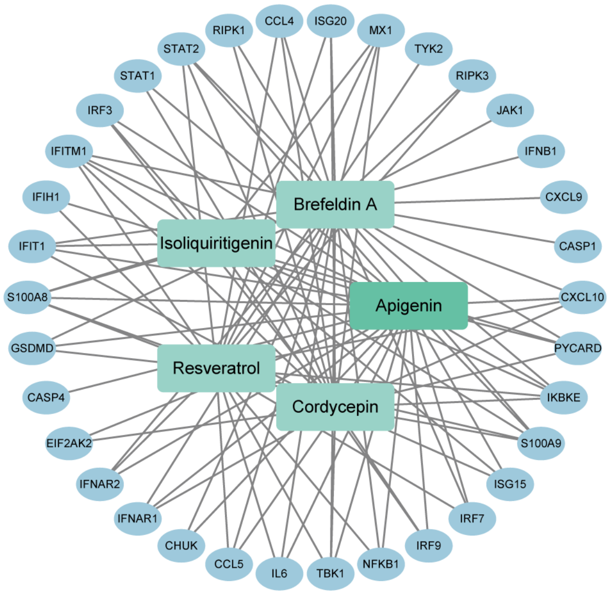

Figure S2. The "ingredient-target" network diagram (generated by Cytoscape software (3.9.1)).

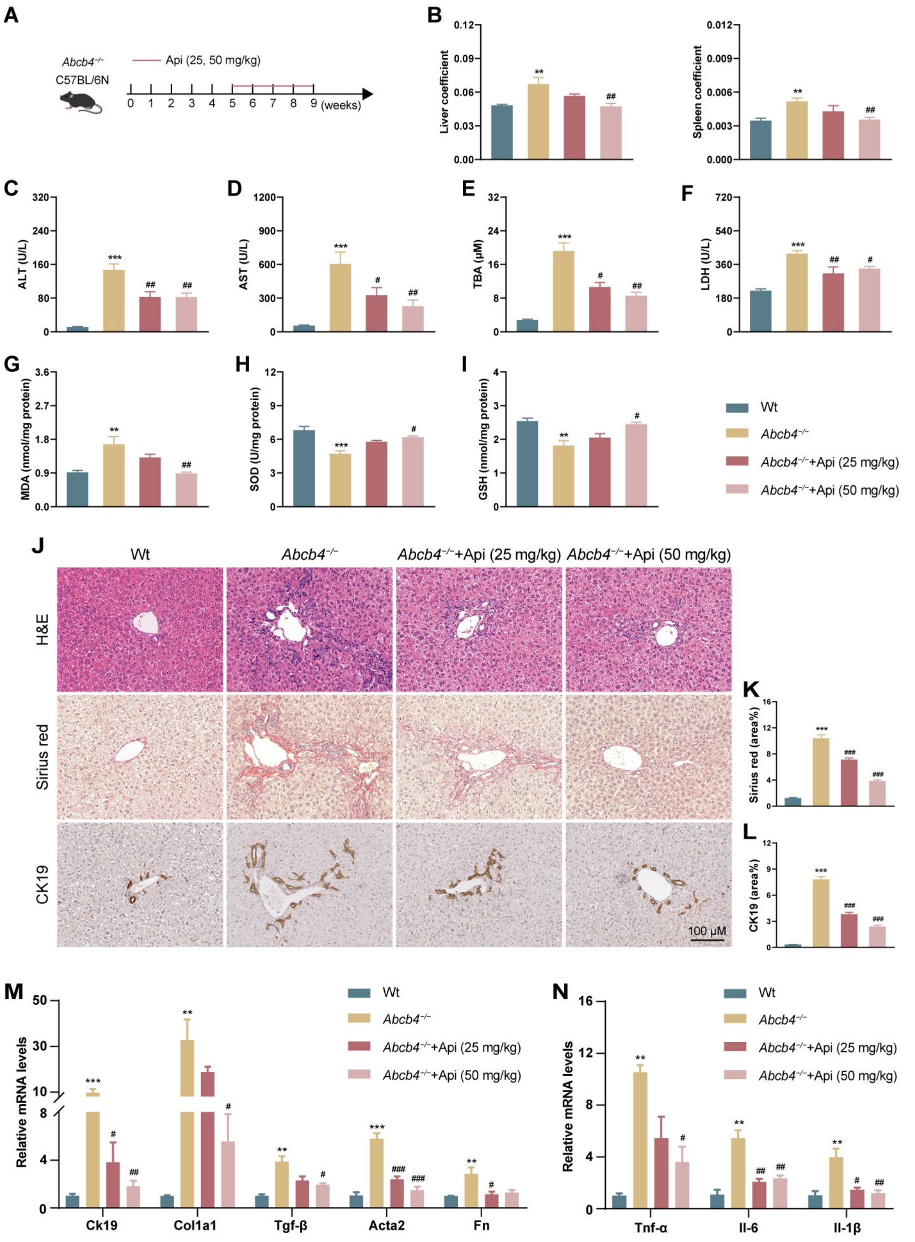

Figure S3. **Effects of Api on liver fibrosis in *Abcb4-/-* mice**. **(A)** The schematic diagram of mice experimental design. **(B)** Liver coefficient and spleen coefficient. Serum levels of ALT **(C)**, AST **(D)**, TBA **(E)** and LDH **(F)**. Hepatic levels of MDA **(G)**, SOD **(H)**, GSH **(I)**. **(J)** H&E staining, Sirius red staining, IHC staining of CK19 in liver sections (scale bar = 100 μm). **(K,L)** Quantification of positive area for Sirius red and IHC staining. **(M)** The mRNA levels of *Ck19*, *Col1a1*, *Tgf-β*, *Acta2*, *Fn* in liver tissues. **(N)** The mRNA levels of *Tnf-α*, *Il-6*, and *Il-1β* in liver tissues. *Hprt1* was used as an internal reference. $^{**}p < 0.01$, $^{***}p < 0.001$ *vs.* the Wt group, $^{\#}p < 0.05$, $^{\#\#}p < 0.01$, $^{\#\#\#}p < 0.001$ *vs.* the *Abcb4-/-* group (n = 6).

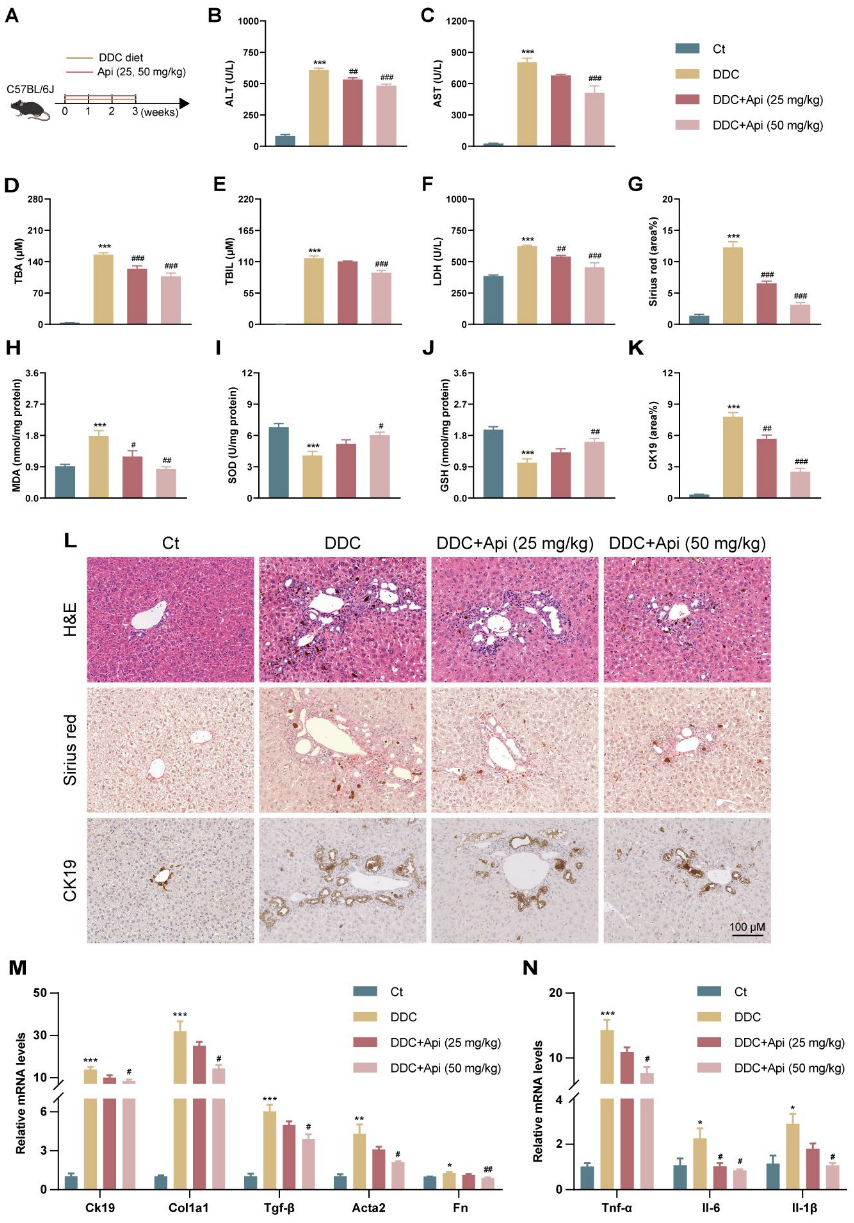

Figure S4. **Effects of Api on DDC-induced hepatic fibrosis in mice**. **(A)** The schematic diagram of mice experimental design. Serum levels of ALT **(B)**, AST **(C)**, TBA **(D)**, TBIL **(E)** and LDH **(F)**. **(G)** Quantification of positive area for Sirius red staining. Hepatic levels of MDA **(H)**, SOD **(I)**, GSH **(J)**. **(K)** Quantification of positive area for IHC staining. **(L)** H&E staining, Sirius red staining, IHC staining of CK19 in liver sections (scale bar = 100 μm). **(M)** The mRNA levels of *Ck19*, *Col1a1*, *Tgf-β*, *Acta2*, *Fn* in liver tissues. **(N)** The mRNA levels of *Tnf-α*, *Il-6*, and *Il-1β* in liver tissues. *Hprt1* was used as an internal reference. $^{*}p < 0.05$, $^{**}p < 0.01$, $^{***}p < 0.001$ *vs.* the Ct group, $^{\#}p < 0.05$, $^{\#\#}p < 0.01$, $^{\#\#\#}p < 0.001$ *vs.* the DDC group (n = 6).

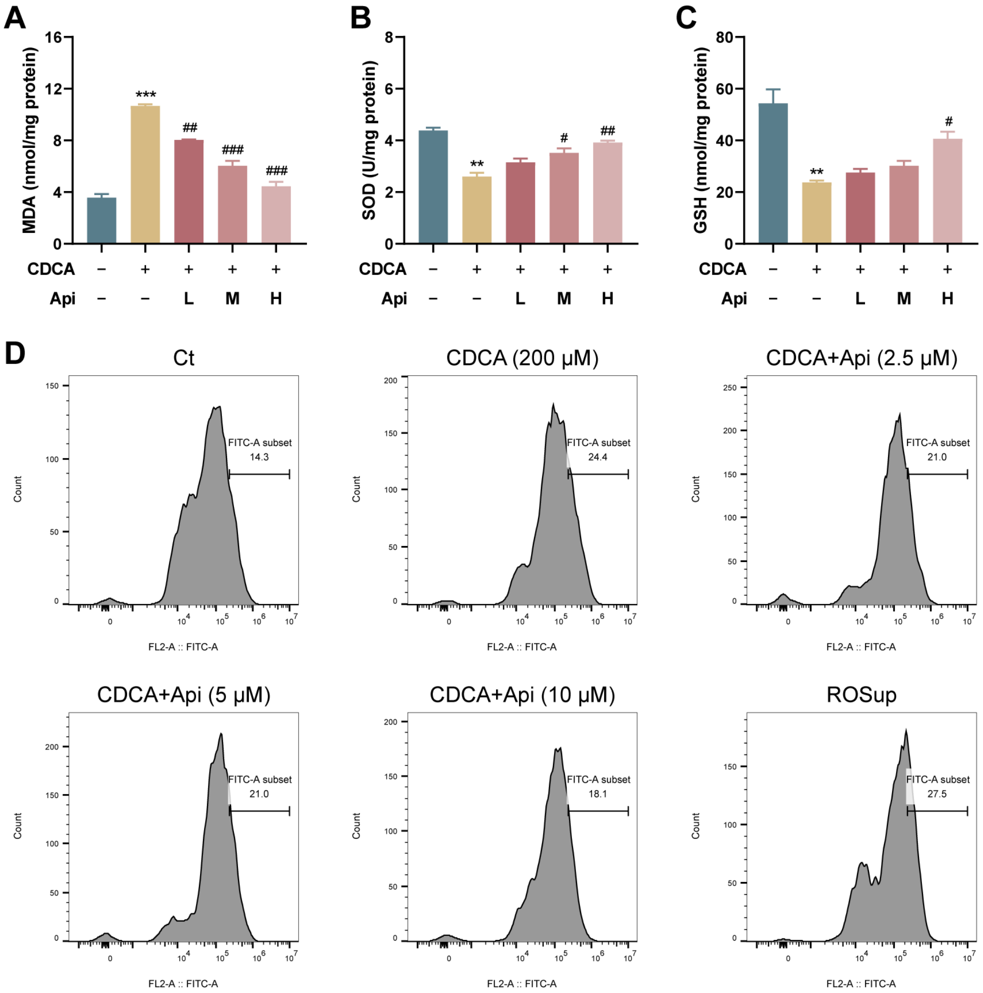

Figure S5. Effects of Api on oxidative stress in CDCA-stimulated HIBEC cells. Cellular levels of MDA **(A)**, SOD **(B)**, GSH **(C)**. **(D)** Cellular ROS detected by flow cytometry. $^{**}p < 0.01$, $^{***}p < 0.001$ *vs.* the Ct group, $^{\#}p < 0.05$, $^{\#\#}p < 0.01$, $^{\#\#\#}p < 0.001$ *vs.* the CDCA group (n = 3).

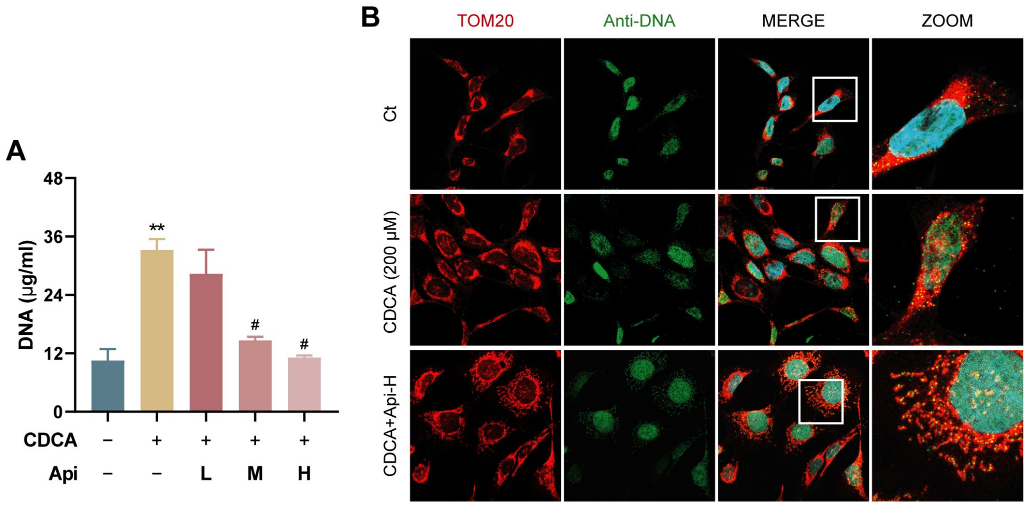

Figure S6. Effects of Api on mtDNA release in CDCA-stimulated HIBEC cells. (A) Profiles of DNA release into the extracellular space by detecting DNA concentration in cell culture medium. **(B)** Fluorescence images of dual of TOM20 (red) and anti-DNA (green) staining for HIBEC cells (nuclei were stained using DAPI (blue), magnification, ×100), white square indicates area displayed in zoom panel. $^{**}p < 0.01$ *vs.* the Ct group, $^{\#}p < 0.05$ *vs.* the CDCA group (n = 3).

Supplementary Table. S1. Primer sequences.

Gene name	Primer sequence	
	F	R
Mouse *Ck19*	GAGCAGATCCAGATAAGCAAGA	GTGACAGCTGGACTCCATAAC
Mouse *Col1a1*	TGAACGTGGTGTACAAGGTC	CCATCTTTACCAGGAGAACCAT
Mouse *Tgf-β*	GACCTCAAGAGCTCTAACATCC	GTCATCCACAGACAGAGTAGG
Mouse *Acta2*	GCGTGGCTATTCCTTCGTGACTAC	TCAGGCAGTTCGTAGCTCTTCTCC
Mouse *Fn*	CTATAGGATTGGAGACACGTGG	CTGAAGCACTTTGTAGAGCATG
Mouse *Il-1β*	AATCTCACAGCAGCACATC	AGCAGGTTATCATCATCATCC
Mouse *Il-6*	TCTGCAAGAGACTTCCATCCAG	ATAGACAGGTCTGTTGGGAGTG
Mouse *Tnf-α*	GTCCCCAAAGGGATGAGAAGT	TTTGCTACGACGTGGGCTAC
Human *Ifnb1*	TGGCTGGAATGAGACTATTGTT	GGTAATGCAGAATCCTCCCATA
Human *Cxcl10*	CTCTCTCTAGAACTGTACGCTG	ATTCAGACATCTCTTCTCACCC
Human *Ifit1*	CAGCCAAGTTTTACCGAAGAAA	GATCATTTGTGCCTTGTAGCAA
Human *Tnf-α*	CGAGTCTGGGCAGGTCTAC	GGGAGGCGTTTGGGAAGG
Human *Il-6*	CAGATTTGAGAGTAGTGAGGAAC	CGCAGAATGAGATGAGTTGTC
Human *Ccl2*	ACCAGCAGCAAGTGTCCCAAAG	TTTGCTTGTCCAGGTGGTCCATG

Supplementary Table. S2. Scoring results based on virtual screening of TCM transcriptome databases.

Number	Ingredient	Up		Down		Score
		ITCM	HERB	ITCM	HERB	
1	Apigenin		6	5	18	17
2	Cordycepin	1	5	9	9	12
3	Resveratrol		4		16	12
4	Isoliquiritigenin			10		10
5	Brefeldin A		9		18	9
6	Liquiritigenin	1		7	3	9
7	Britanin		1		9	8
8	Eupalinolide A	2		10		8
9	Liguiritigenin-7-O-D-apiosyl-4'-O-D-glucoside			7		7
10	Isoliquiritin	1		8		7
11	Senecionine		7		14	7
12	Ginkgolic acid				7	7
13	withaferin a		1		7	6
14	Ginsenoside rb2	2	1	2	7	6
15	Muscone			1	5	6
16	Paeoniflorin			6		6
17	Cardamonin	1		7		6
19	Trifolin			5		5
20	Corydaline			5		5
21	Glycyrrhetinic acid			5		5
22	Patchouli alcohol			5		5
23	Phloridzin		1		6	5
24	Griffonilide	2		7		5
25	Hesperidin	9		11	2	4
26	Schizandrin B	1		5		4
27	Cycloastragenol			4		4
28	Isochlorogenic acid a			4		4
29	Taxol		9		13	4
30	Ginsenoside re	2	1	4	3	4
31	Progesterone		8		12	4
32	Digoxin		8		12	4
33	Parthenolide				4	4
34	Protocatechuic aldehyde				4	4
35	Arctigenin		1		5	4
36	Norisoboldine	1		5		4
37	Magnolin			4		4
38	Alkannin	3		2	4	3
39	Hyperoside	8		8	3	3

40	Oleanolic acid	1		3	1	3
41	Diosgenin	1		4		3
42	Corynoxeine			3		3
43	Isoflavone			3		3
44	Cannabidiol		8		11	3
45	Linolenic acid		2		5	3
46	Linalool		1		4	3
47	Cinnamaldehyde	1			4	3
48	Toddalolactone			3		3
49	(+)-Corynoline	1		4		3
50	Pseudolaric Acid B	5		8		3
51	Vitexin	1		3		2
52	Quercetin	1	6	2	7	2
53	Cornuside I			2		2
54	Fargesin			2		2
55	Rosmarinic acid			2		2
56	Luteolin		9	2	9	2
57	Apigenin-7-Glucuronide	1		3		2
58	Ginsenoside Rb3	3		2	3	2
59	Lcaritin	2		4		2
60	Psoralidin			2		2
61	Tetrahydrocoptisine			2		2
62	Oroxylin A 7-O-β-D-Glucuronide			2		2
63	Salvianolic acid B	2		4		2
64	Humulene			2		2
65	Isoborneol		2		4	2
66	Ginsenoside rc		1		3	2
67	Sauchinone				2	2
68	Bacopaside i		1		3	2
69	Tanshinone iia				2	2
70	Rottlerin		1		3	2
71	Sanguinarine				2	2
72	Tectoridin	1		3		2
73	Dehydrodiisoeugenol	1		3		2
74	Tanshinone IIA-sulfonic sodium	1		3		2
75	Atractylodin	2		4		2
76	Panaxtriol			2		2
77	Puerarin	2		2	1	1
78	Irisflorentin	8		9		1
79	Baicalin	4		5		1
80	Schisandrin A	2		3		1
81	Kaempferol			1		1

82	Rotundifuran	8		9		1
83	Icariin			1		1
84	Baohuoside I	1		2		1
85	Corylin			1		1
86	Bakuchiol			1		1
87	Synephrine			1		1
88	Soyasaponin I	2		3		1
89	Imperatorin	1	2	2	2	1
90	Oridonin		2		3	1
91	Stigmasterol				1	1
92	Harmine		1		2	1
93	Pinocembrin			1		1
94	Schisantherin C			1		1
95	Capsaicin			1		1
96	Mesaconitine			1		1
97	Racanisodamine	3		4		1
98	Columbianadin	2		3		1
99	Pseudoprotodioscin			1		1
100	Indirubin			1		1
101	Panaxadiol			1		1
102	Polygalic acid			1		1
103	Prunasin	9		9		0
104	Wogonin	1		1		0
105	Dioscin		1		1	0
106	Myricetin					0
107	Formononetin	1		1		0
108	Gypenoside XVII	1		1		0
109	Safflomin A	1		1		0
110	Tectorigenin	1		1		0
111	6-Gingerol	3		3		0
112	Batatasin I	2		2		0
113	Embelin		5		5	0
114	Nitidine chloride		3		3	0
115	Artemisinin		1		1	0
116	Menthol		6		6	0
117	Borneol		1		1	0
118	Gedunin		1		1	0
119	Berberine		3		3	0
120	Epigallocatechin 3-gallate		1		1	0
121	Santonin		1		1	0
122	Sennoside A		1		1	0
123	Saikosaponin D		1		1	0
124	DL-Isoborneol	2		2		0
125	Tilianin	3		3		0

126	Tussilagone	2		2		0
127	Ethyl 4-methoxycinnamate	1		1		0
128	Polydatin	1		1		0
129	Shanzhiside methylester	1		1		0
130	Plantamajoside	1		1		0
131	Forsythoside B	2		2		0
132	Engeletin	1		1		0
133	Vitexicarpin	3		3		0
134	Cynaroside	1		1		0
135	Evodiamine					0
136	Honokiol	3		2		-1
137	Pseudoephedrine	2		1		-1
138	Isorhamnetin	3		2		-1
139	Rhein	1				-1
140	Salicylic acid	1				-1
141	Scutellarin	2		1		-1
142	Schisanhenol	3		2		-1
143	Ononin	2		1		-1
144	Genkwanin	3		2		-1
145	Nobiletin	1				-1
146	Limonin	1				-1
147	A-Terpineol	1				-1
148	Silybin		1			-1
149	Alantolactone	3	9	10	1	-1
150	Benzoylaconitine		1			-1
151	Ginsenoside Rb1		3		2	-1
152	Lobetyolin		1			-1
153	Saikosaponin A		1			-1
154	Cinnamic acid	1	2		2	-1
155	Farnesol		1			-1
156	Daphnetin	2		1		-1
157	Rutin	1				-1
158	Lristectorin A	2		1		-1
159	Pachymic acid	2		1		-1
160	Cyclovirobuxine	1				-1
161	Purpurin	2		1		-1
162	Rhoifolin	1				-1
163	Dipsacoside B	2		1		-1
164	Palmatine hydrochloride	1				-1
165	8-O-Acetylharpagide	1				-1
166	Daphnoretin	1				-1
167	Germacrone	3		2		-1
168	5-O-Methylvisammioside	1				-1
169	Artemisinine	1				-1

170	6,7-Dihydroxycoumarin	1				-1
171	Baicalein	1	2	1	1	-1
172	Isofraxidin	7		5		-2
173	Gentiopicrin	3		1		-2
174	Astragaloside II	2				-2
175	Ferulic acid	1	1			-2
176	Emodin		2			-2
177	Neochlorogenic acid	2				-2
178	Isochlorogenic acid C	2				-2
179	Caffeic acid	2				-2
180	3,4-Dihydroxybenzoic acid	2				-2
181	Schizandrol A	3		1		-2
182	Salvianic acid A	2				-2
183	Tanshinone I	4		2		-2
184	Cryptotanshinone	3		1		-2
185	Albiflorin	2				-2
186	Tetrahydropalmatine	2				-2
187	Quinic acid	2				-2
188	Isoirigenin	2				-2
189	Ephedrine	3		1		-2
190	Succinic acid	2				-2
191	Berberine hydrochloride		3		1	-2
192	Bilobalide	2	1		1	-2
193	Gastrodin		3		1	-2
194	Ginkgolide B		3		1	-2
195	Monocrotaline		5		3	-2
196	Endo-Borneol	2				-2
197	Shionone	2				-2
198	8-Gingero	2				-2
199	Asarinin	2				-2
200	Complanatuside	2				-2
201	Sarsasapogenin	2				-2
202	Pectolinarin	4		2		-2
203	Wedelolactone	4		2		-2
204	Orientin	2				-2
205	Praeruptorin B	7		5		-2
206	Vaccarin	2				-2
207	Sesamin	1	1			-2
208	Madecassoside	2				-2
209	Amygdalin	8		5		-3
210	Glycyrrhizic acid	12		6	3	-3
211	Verbascoside	2	2		1	-3
212	Liquiritin	6		3		-3
213	Alisol b 23-acetate	6		3		-3

214	Isochlorogenic acid B	3				-3
215	4-Dicaffeoylquinic acid	3				-3
216	Quercitrin	3				-3
217	Quercetin-3-O-Glucuronide	3				-3
218	Ginsenoside Rd	4	1		2	-3
219	Notoginsenoside R2	4		1		-3
220	Neohesperidin	3				-3
221	Beta-Elemene	3				-3
222	1-O-caffeoylquinic acid	5		2		-3
223	Alisol O	3				-3
224	Naringin	3				-3
225	Senkyunolide H	4		1		-3
226	(-)-Caryophyllene	4		1		-3
227	Ginsenoside Rg1		4		1	-3
228	Tetrandrine	5		2		-3
229	Ergosterol	3				-3
230	10-Gingerol	3				-3
231	Cynarin	5		2		-3
232	Fangchinoline	5		2		-3
233	Saikosaponin B	3				-3
234	Harpagide	3				-3
235	Chrysophanol	5		2		-3
236	Spinosin	4		1		-3
237	Nodakenin	6		3		-3
238	Curdione	6		3		-3
239	Sophoridine	3				-3
240	Notopterol	3				-3
241	Indigo	3				-3
242	Swertiamarin	4				-4
243	Turicine	4				-4
244	Ginsenoside Ro	4				-4
245	Licochalcone A		9		5	-4
246	Plumbagin		4			-4
247	Celastrol		9		5	-4
248	Atractylenolide-1	4				-4
249	Dihydrocapsaicin	6		2		-4
250	Pedunculoside	4				-4
251	Tenuifolin	4				-4
252	Asiaticoside	5		1		-4
253	Schaftoside	4				-4
254	Arbutin	4				-4
255	Isoastragaloside	11		6		-5
256	Triptolide		10		5	-5
257	Pilocarpine		7		2	-5

258	Ruscogenin	6		1		-5
259	Polygalaxanthone III	6		1		-5
260	Liriope muscari baily saponins C	5				-5
261	Isoquercitrin	5				-5
262	Calycosin7-O-β-D-glucopyranoside	12		6		-6
263	Syringin	7		1		-6
264	Ginsenoside Rf	6				-6
265	Curcumol	8		2		-6
266	Liensinine	8		2		-6
267	Curcumin	4	15	6	6	-7
268	Calycosin	13		6		-7
269	Cantharidin	14		7		-7
270	Eleutheroside E	7				-7
271	Gallic acid	1	10	3	1	-7
272	Ursolic acid		16	2	7	-7
273	Bufalin		8		1	-7
274	Daidzein	2	11		6	-7
275	Bruceine D		13		5	-8
276	Genistein		13		5	-8
277	Isoalantolactone		9			-9
278	Rotenone		12		3	-9
279	Leonurine hydrochloride	10		1		-9
280	Vincristine		15		4	-11
281	Narciclasine		14		2	-12
282	Isoginkgetin		13			-13
283	Maslinic acid		22		1	-21
